# Research on the impact of digital upgrade of sports event industry on value chain reconstruction

**DOI:** 10.3389/fspor.2026.1859039

**Published:** 2026-09-25

**Authors:** Lide Su, ZhiXiang Wen, Te Bu

**Affiliations:** College of Physical Education, Hunan Normal University, Changsha, China

**Keywords:** digital upgrading, organizational boundary, platform coordination, sports event industry, value chain restructuring

## Abstract

**Background:**

Digital upgrading is reshaping how sport event industries organize value creation, coordination, and commercialization. However, limited attention has been paid to how digital upgrading reconstructs organizational boundaries and value chains.

**Methods:**

This study adopts a sequential mixed-method design combining multiple-case analysis and quantitative testing. Based on 12 sport event cases and 28 semi-structured interviews, the study identifies restructuring paths through qualitative coding and further examines structural effects using social network analysis, event-study estimation, and difference-in-differences models.

**Objectives:**

The study examines how digital upgrading changes organizational boundaries, reallocates value activities, and drives value chain restructuring in the sport event industry.

**Results:**

Four restructuring paths are identified, including content-led, platform-driven, service-integration, and data-asset extension pathways. Digital upgrading is associated with increased network density from 0.31 to 0.39, significant improvements in value chain restructuring (DID coefficient = 0.082, *p* < 0.001), sponsorship conversion (0.058, *p* < 0.01), and data monetization (0.064, *p* < 0.01). The effects are stronger in events with higher platform capability and richer data assets.

**Conclusion:**

Digital upgrading represents a governance and value-chain restructuring process rather than a simple technological adoption process. By linking digital capabilities, organizational boundary adjustment, ecosystem coordination, and value redistribution, this study advances understanding of digital transformation mechanisms in sport event industries.

## 1 Introduction

The deepening of the digital economy is reshaping industrial organization and value creation, and the sport event industry is entering a new stage of structural adjustment characterized by data-driven operations (1), platform-based coordination (2), and scenario-based value extension (3). Traditionally, the value chain of sport events has revolved around event planning (4), content production, media distribution, ticketing, sponsorship development (5), and on-site services, with its operation largely based on linear division of labor, hierarchical coordination (6), and relatively stable transactional relationships (7). With the widespread application of cloud computing, big data, artificial intelligence, mobile internet, and platform technologies in event operations, the connections among event organizers, digital platforms (8), media agencies, ticketing providers, sponsors, and data service firms have changed substantially (9). As a result, the value center, revenue sources, and coordination mechanisms of the industry are undergoing profound transformation (10). Digitalization is no longer a technical supplement to existing business processes, but is increasingly becoming a major force driving industrial upgrading and business model renewal in the sport event sector (11).

From a practical perspective, digital upgrading in the sport event industry is shaped by both market demand and governance constraints (12). Intensified market competition, increasingly personalized consumer demand, and the growing need for fine-grained evaluation of sponsorship returns have pushed event organizations to improve content reach (13), enhance service experiences, and strengthen commercialization through digital technologies. At the same time, the sport event industry is marked by a high degree of cross-organizational coordination (14), complex resource dependence, and the coexistence of public and commercial attributes (15). Digital transformation in this context is therefore not merely an internal technological adjustment, but a broader process of organizational change embedded in specific governance arrangements (16). Event organizers must not only pursue efficiency gains and revenue expansion, but also respond to practical issues such as platform dependence, data ownership, inter-organizational governance, and value distribution (17). In this sense, digital upgrading in the sport event industry should be understood as the combined outcome of technological diffusion, organizational restructuring, and governance adaptation.

In practice, digital technologies have already become deeply embedded across multiple key segments of the sport event value chain, showing a clear shift from isolated application to systemic integration (18). In content production, intelligent capture, data visualization, immersive communication, and multi-platform distribution have increased the reproducibility and circulation capacity of event content. In marketing and distribution, platform algorithms, user profiling, and precision targeting have significantly altered audience reach and traffic allocation (19). In ticketing and service delivery, online ticketing systems, dynamic pricing, membership systems (20), and digital identity authentication have promoted the integration of transaction processes and user services (21). In sponsorship conversion, data monitoring and interactive operations have strengthened the measurable evaluation and sustained activation of sponsorship rights (22). In data monetization, new services and products built around event data, user data, and behavioral data are expanding the commercial boundaries of sport events (23). These developments indicate that digital upgrading not only improves the operational efficiency of individual segments, but also redefines the division of responsibilities, interface relationships, and collaborative patterns among participating actors.

Existing research on sport event digitalization and industrial upgrading has generated a meaningful body of knowledge. Some studies have examined how digital technologies empower event communication, fan engagement, and service innovation, emphasizing their positive effects on audience experience and operational efficiency (24). Other studies, from the perspective of platform organization, have explored the intermediary role of digital platforms in resource integration, traffic distribution, and commercial connection, arguing that platform firms are becoming key coordinators within sport event value networks (25). A further stream of research has focused on event value creation and industrial collaboration, paying attention to the linkages among event branding, sponsorship performance, media dissemination, and derivative development (26). Overall, this literature provides an important foundation for understanding the practical logic of digital development in sport events. However, much of the existing work remains concentrated on the effects of technology adoption, business model innovation, or the optimization of individual value activities, while offering limited explanation of how digital upgrading triggers structural transformation of the value chain as a whole.

More importantly, insufficient attention has been paid to the internal linkages among digital technology, organizational boundaries, and the reconfiguration of value activities. Digital upgrading does not automatically lead to value chain restructuring (27). Its structural effect depends on how organizational boundaries are redefined after technological adoption, how control rights, professional specialization, and collaborative relationships are adjusted among actors, and how the resulting recombination of value activities reshapes power structures and revenue distribution within the industry chain. In other words, the crucial mechanism through which digital technology produces structural change lies in its capacity to alter the boundary arrangements among event organizers, platforms, service providers, content producers, and sponsors, thereby driving the reallocation of content production, marketing and distribution (28), ticketing services, sponsorship conversion, and data monetization. Current research on this process remains fragmented. A systematic analytical framework that explains value chain restructuring in the sport event industry from the perspective of organizational boundary adjustment is still lacking, as is empirical evidence that combines qualitative path identification with structural quantitative testing.

Although existing studies have documented the operational benefits of digital technologies in sport events, less attention has been paid to the theoretical mechanisms through which digital upgrading generates structural changes in industry organization. This study draws on multiple complementary theoretical perspectives to explain why digital upgrading is significantly associated with value chain restructuring and provides evidence consistent with its role in driving structural change. Organizational boundary theory suggests that firms adjust the scope of internal control and external coordination when changes in information accessibility, transaction efficiency, and capability distribution alter the relative advantages of different governance arrangements. In the sport event context, digital technologies reduce coordination costs while increasing the strategic importance of data, platforms, and user relationships, thereby encouraging selective boundary contraction, external collaboration, and ecosystem embedding.

Transaction Cost Economics further explains why digital upgrading changes governance choices. Traditional sport event value chains rely heavily on sequential transactions among organizers, media firms, sponsors, and service providers. Digital platforms and shared data infrastructures reduce information asymmetry, monitoring costs, and coordination barriers, making new forms of networked collaboration economically feasible. However, activities involving strategic assets, brand control, or critical user relationships may remain internalized because ownership and control generate additional value. This theoretical logic explains why digital upgrading produces both internal capability consolidation and external ecosystem expansion.

Platform ecosystem theory provides insight into why some restructuring paths become platform-centered. Digital platforms create multi-sided coordination mechanisms by connecting event organizers, audiences, sponsors, and service providers. Events with stronger platform capabilities are more likely to shift toward platform-driven restructuring because platforms become central nodes for traffic allocation, user interaction, and commercial conversion. Dynamic capability theory further explains why restructuring outcomes differ across events. Digital upgrading requires organizations to sense emerging opportunities, integrate digital resources, and reconfigure existing activities. Events with stronger data capabilities, commercialization experience, and organizational adaptability are more capable of converting digital inputs into deeper value chain transformation.

Based on these theoretical perspectives, Although existing studies have provided valuable insights into the application of digital technologies in sport events, three limitations remain. First, current research largely focuses on operational improvements, such as fan engagement, service efficiency, and communication enhancement (29), while paying insufficient attention to how digital upgrading reshapes the structure of value creation across the industry chain. Second, previous studies have rarely examined the organizational mechanisms underlying digital restructuring, particularly how digital technologies alter organizational boundaries, ecosystem relationships, and the allocation of value activities among multiple actors. Third, empirical research has provided limited explanation of why similar digital initiatives generate different restructuring outcomes across sport events with different resource conditions and governance arrangements. Addressing these gaps, this study conceptualizes digital upgrading as a process through which digital capabilities progressively reshape organizational boundaries, value activities, and ecosystem relationships. Drawing on organizational boundary theory, platform ecosystem theory, Transaction Cost Economics, and dynamic capabilities perspectives, the study develops an integrated framework linking digital upgrading, boundary adjustment, value activity reconfiguration, and value chain restructuring. A sequential mixed-method design is adopted, combining comparative case analysis with quantitative examination of network structures, value flows, and restructuring effects (20).

Specifically, this study addresses three research questions: (1) How does digital upgrading reshape organizational boundaries and value activities in the sport event industry? (2) What restructuring paths emerge from different configurations of digital capabilities and ecosystem conditions? (3) To what extent is digital upgrading associated with measurable changes in network structure, value distribution, and industry performance? By answering these questions, this study contributes to digital transformation research by extending existing theories into the sport event context and provides practical insights into governance and value creation in digitally mediated sport ecosystems. To answer these questions, the study develops an analytical logic in which digital technology affects organizational boundaries, organizational boundaries drive the reconfiguration of value activities, and such reconfiguration ultimately leads to value chain restructuring. Building on this framework, the study combines comparative multiple-case analysis, network-based structural mapping, and causal identification strategies. The contribution of this research is threefold. At the theoretical level, it moves the study of sport event digitalization beyond technology application and toward industrial organizational restructuring, thereby deepening understanding of the mechanisms of value chain transformation. At the analytical level, it establishes an integrated explanatory framework linking digital technology, organizational boundaries, and value activity reconfiguration, offering a more structural perspective for research on digital transformation in sport events. At the empirical level, by combining cross-case comparison with quantitative analysis, it identifies the specific pathways, triggering conditions, and performance consequences of value chain restructuring, and thus provides more actionable evidence for digital governance and business model optimization in the sport event industry.

To improve conceptual clarity, this study distinguishes digital upgrading from several related but non-equivalent concepts. Digitalisation generally refers to the process through which digital technologies are adopted and embedded into existing activities, such as the use of digital platforms, data systems, and online service tools to improve operational efficiency. Digital transformation represents a broader organisational and strategic change process in which digital technologies reshape business models, governance structures, and value creation mechanisms. Building on these concepts, digital upgrading is defined in this study as a capability-enhancement process through which sport event organisations progressively integrate digital technologies, data resources, and platform-based mechanisms into core value activities, thereby improving coordination efficiency, expanding commercial capabilities, and reshaping value chain structures. Therefore, digital upgrading is not used as a synonym for technology adoption or general digital transformation, but as a specific analytical perspective focusing on how incremental digital capability enhancement generates structural changes in organisational boundaries and value activities within sport event industries.

## 2 Methods

### 2.1 Research design and analytical framework

This study adopts a sequential mixed-method design that integrates comparative multiple-case analysis with quantitative testing. The research proceeds in two stages. The first stage uses multiple-case comparison to identify how digital upgrading reshapes inter-organizational relationships and reallocates value activities across the sport event industry chain. This stage is intended to uncover the main restructuring paths, the changing roles of core actors, and the conditions under which digital technologies produce deeper structural change. The second stage moves from mechanism identification to empirical verification. Based on the qualitative findings, the study constructs measurable indicators of digital upgrading and value chain restructuring, and then applies network-based measures and econometric tests to examine whether digital upgrading is associated with structural changes in coordination patterns, value capture, and key performance indicators. This design is appropriate because value chain restructuring in the sport event industry is both processual and structural. It requires qualitative evidence to capture organizational adjustment and quantitative evidence to assess whether such adjustment leads to observable changes in industrial relations and performance.

The analytical framework is built around the logic linking digital technology, organizational boundaries, value activity reconfiguration, and value chain restructuring. In the sport event industry, digital upgrading is not treated as the mere application of new tools to existing operations, but as an organizational process that alters how event organizers, platforms, service providers, sponsors, and data intermediaries interact. The focus is placed on how the introduction and deepening of digital technologies change the boundaries of coordination, the allocation of functions, and the structure of value creation across key segments such as content production, marketing and distribution, ticketing, sponsorship conversion, and data monetization. Accordingly, value chain restructuring is understood as a structural outcome reflected in changes in network connectivity, actor centrality, inter-firm collaboration, and the distribution of value capture across industry segments.

From the perspective of networked production and platform coordination, digital upgrading tends to strengthen interdependence among key actors in the sport event industry. As digital systems improve information transparency, reduce coordination costs, and enable real-time interaction across organizational interfaces, the traditional linear structure of content creation, distribution, sales, and commercial conversion is gradually replaced by a more connected and platform-mediated arrangement. In this process, core nodes in the value chain are more likely to form denser collaborative ties, and the network itself becomes more integrated rather than segmented. Based on this reasoning, the study proposes Hypothesis 1:

H1: The deeper the level of digital upgrading, the stronger the collaborative linkages among core nodes in the sport event industry chain, and the more likely the value chain network is to evolve from a linear division-of-labor structure toward a platform-coordinated collaborative structure.

From the perspective of organizational boundary adjustment, digital upgrading changes not only the efficiency of existing activities but also the scope and control of those activities. The diffusion of digital platforms, data systems, and integrated service architectures may lead event organizers to internalize some high-value functions, outsource standardized tasks, or establish new forms of modular collaboration with external partners (30). As organizational boundaries are redefined, key value activities such as content production, marketing distribution, and ticketing services are reallocated across actors with different resource endowments and capabilities. This reallocation is likely to shift value capture toward activities with higher data intensity, stronger user linkage, and greater monetization potential. Based on this reasoning, the study proposes Hypothesis 2:

H2: Digital upgrading reshapes organizational boundaries, drives the reconfiguration of key value activities including content production, marketing distribution, and ticketing services, and improves value capture in high value-added segments.

From the perspective of conditional heterogeneity, the effects of digital upgrading are unlikely to be uniform across all types of sport events. Events with stronger platform capabilities, richer data assets, and more developed commercialization scenarios are better positioned to convert digital inputs into sustainable revenue channels (31). In these contexts, digital upgrading can more effectively enhance sponsor activation, audience targeting, and data-based service provision, thereby generating stronger improvements in sponsorship conversion efficiency and data monetization capacity. Such events are therefore more likely to experience deeper and more comprehensive value chain restructuring. Based on this reasoning, the study proposes Hypothesis 3:

H3: In sport events with stronger platform capability, richer data assets, and more mature commercialization scenarios, digital upgrading generates greater improvements in sponsorship conversion efficiency and data monetization, thereby making deeper value chain restructuring more likely.

The research object of this study is the digital upgrading of the sport event industry and its impact on value chain restructuring. Digital upgrading is defined here as the process through which sport event organizations embed digital infrastructures, data systems, and platform-based operating mechanisms into core business activities, thereby changing patterns of production, coordination, service delivery, and commercialization. Its boundary is limited to industry-level transformations directly associated with event operation and related commercial linkages, rather than broader digital development in the general sports sector (32). The main observational dimensions include digital embedding in content production, digital transformation of marketing and distribution, digital integration in ticketing and audience services, digital enhancement of sponsorship activation, and the expansion of data-driven monetization activities. These dimensions jointly reflect the extent to which digital technologies alter industrial interfaces and reshape the architecture of value creation in the sport event industry.

In this study, digital upgrading serves as the core explanatory concept and is operationalised through five dimensions: digital content production, platform-based distribution, digital ticketing and service integration, data-enabled sponsorship activation, and data monetisation capability. These dimensions capture the extent to which digital technologies move from supporting individual operational tasks toward reorganising inter-organisational coordination and value creation processes. Accordingly, digital upgrading represents the measurable pathway through which digitalisation evolves into broader transformation effects at the value chain level. This conceptualisation allows the study to examine not only whether digital technologies are adopted, but also how their deeper integration changes the structure and functioning of the sport event value chain. The analytical framework is grounded in an integrated theoretical perspective combining organizational boundary theory, platform ecosystem theory, Transaction Cost Economics, and dynamic capabilities. Digital upgrading is conceptualized as a capability-enhancement process that changes the cost structure, resource accessibility, and coordination mechanisms of sport event organizations. Organizational boundary adjustment represents the governance response to these changes, determining whether activities are internalized, outsourced, platform-coordinated, or embedded within broader ecosystems. Value activity reconfiguration represents the operational manifestation of these boundary changes, while value chain restructuring represents the resulting transformation in network relationships and value distribution.

The framework also recognizes that restructuring paths are contingent on organizational and market conditions. Platform capability influences whether events move toward platform-centered coordination, data asset maturity affects the feasibility of data-driven commercialization, and commercialization scenarios determine whether digital resources can be converted into economic value. Therefore, digital upgrading does not generate a uniform restructuring outcome but produces differentiated pathways depending on resource conditions, governance choices, and ecosystem environments. The sequential mixed-method design follows an exploratory-to-explanatory logic, in which qualitative findings provide the theoretical and empirical foundation for subsequent quantitative testing. The qualitative phase first identifies how digital upgrading reshapes the sport event value chain through changes in organizational boundaries and value activities. Through cross-case comparison, four restructuring paths are derived, including content-led restructuring, platform-driven restructuring, service-integration restructuring, and data-asset extension restructuring. These paths are then translated into measurable dimensions and analytical variables for the quantitative phase.

Specifically, the qualitative findings informed three aspects of the quantitative design. First, the identification of restructuring paths guided the construction of the value chain restructuring index. The observed changes in content production, marketing distribution, ticketing services, sponsorship conversion, and data monetization were transformed into measurable dimensions of the outcome variable. Second, the boundary adjustment mechanisms identified in the qualitative analysis informed the selection of network indicators, including network density, centrality, and collaborative embeddedness, which capture changes in inter-organizational relationships. Third, the conditions under which different restructuring paths emerged informed hypothesis development and heterogeneity analysis, particularly regarding the roles of platform capability, data assets, and commercialization scenarios. The quantitative models were therefore designed to test whether the restructuring mechanisms identified qualitatively were reflected in observable structural changes. The event-study model examines the dynamic changes surrounding digital upgrading events, while the difference-in-differences model evaluates whether events implementing substantial digital upgrading experience greater changes in restructuring outcomes compared with comparable events. The integration of qualitative and quantitative approaches allows the study to move from identifying restructuring mechanisms to testing their broader empirical patterns.

### 2.2 Case selection and data sources

The study recruited participants using purposive sampling combined with criterion-based sampling. Participants were required to have direct experience with sport event participation or digitalized event services, including athletes, event participants, and users of digital event platforms. Participants without direct experience of digital sport event services were excluded.

A total of 426 questionnaires were distributed, and 384 valid responses were retained after data screening, resulting in a valid response rate of 90.14%. The final sample consisted of 226 male participants (58.85%) and 158 female participants (41.15%), with an average age of 27.6 years (SD = 6.8). Among them, 142 participants (36.98%) had experience participating in professional or large-scale sport events, 156 participants (40.63%) participated in mass-participation events, and 86 participants (22.40%) had experience with digitally enhanced event services such as mobile ticketing, online registration, and platform-based engagement systems. The diversity of participant backgrounds provided empirical support for examining how digital upgrading influences sport event experience and value creation mechanisms. The Athlete Experience Questionnaire (AEQ) was administered to measure participants' perceptions of digitalized sport event experiences. The questionnaire consisted of 18 items across four dimensions, including digital interaction experience (5 items), service accessibility (4 items), information transparency (4 items), and perceived value enhancement (5 items). All items were measured using a five-point Likert scale ranging from 1 (strongly disagree) to 5 (strongly agree). The reliability analysis showed that the overall scale achieved satisfactory internal consistency (Cronbach's *α* = 0.91), with subscale reliability coefficients ranging from 0.84 to 0.89.

The case selection follows a purposive and theoretically informed sampling strategy. Cases are chosen to ensure variation in event type, level of digital upgrading, business model, and data accessibility, so that the comparison can capture different restructuring trajectories and conditions. Variation in event type is necessary because professional leagues, comprehensive sports events, and participatory events differ substantially in organizational complexity, revenue logic, and audience interaction. Variation in digital upgrading level is needed to observe how different degrees of digital embedding influence changes in organizational boundaries and value activities. Variation in business model helps identify whether events with stronger media dependence, ticketing dependence, or sponsorship dependence respond differently to digital transformation. Data accessibility is also a key consideration, as the study requires sufficient information on industrial linkages, operational arrangements, and performance outcomes to support both qualitative comparison and quantitative analysis.

The sample is composed of several categories of sport events with contrasting organizational and commercial characteristics. The first group includes leading professional events that usually possess relatively mature media systems, stable commercial resources, and stronger digital operating capabilities. The second group includes comprehensive or multi-sport events, which involve more complex governance structures and broader stakeholder coordination. The third group includes road running events and other mass participation events, where participant services, ticketing substitutes, digital engagement, and community-based commercialization often play a more prominent role. The comparative logic is not to rank these event types, but to examine how differences in organizational complexity, platform dependence, and monetization structure shape the pathways and depth of value chain restructuring under digital upgrading.

The study draws on multiple sources of evidence in order to improve construct validity and reduce the limitations of single-source inference. Platform operation data are used to capture traffic, user interaction, distribution reach, and digital engagement. Ticketing and sponsorship materials provide information on sales mechanisms, service integration, sponsorship activation, and commercial conversion. Annual reports, corporate disclosures, and official announcements are used to identify major digital initiatives, strategic adjustments, and structural changes in industry relationships. Semi-structured interviews with event organizers, platform operators, service providers, and industry experts are used to clarify decision logic, boundary adjustment, and activity reallocation that may not be directly observable in public data. The questionnaire and interviews covered only sport event experiences, service perceptions, and industry-level operational and commercial topics, and did not involve any personal information, health data, or other sensitive or ethically relevant content. Secondary textual materials, including industry reports, media coverage, and event documents, are used to supplement the evidence base and support cross-validation. The use of diverse data sources allows the study to reconstruct the process of digital upgrading with greater analytical depth and empirical reliability.

### 2.3 Variable definition and measurement

To connect the qualitative and quantitative stages, the study defines a set of key variables that reflect the degree of digital upgrading, the extent of value chain restructuring, and the structural changes occurring across major industry segments. The explanatory variable is the level of digital upgrading. This variable captures the extent to which sport events adopt and integrate digital technologies into their operational and commercial systems. It is measured through a composite index covering digital content production, platform-based distribution, digital ticketing and service systems, data-enabled sponsorship activation, and data monetization infrastructure. The outcome variable is the degree of value chain restructuring. This variable reflects structural changes in inter-organizational coordination, actor positioning, and value allocation within the sport event industry chain. It is measured through indicators such as network density, changes in node centrality, shifts in collaboration patterns, and variation in value capture across key segments.

To better identify the internal structure of value chain transformation, the study further develops several dimension-specific indicators. Content production restructuring refers to changes in the organization, modularization, and digital circulation of event-related content. Marketing and distribution restructuring captures the transition from traditional channel-based promotion to platform-driven and data-targeted distribution. Ticketing service restructuring reflects the integration of ticketing, membership, identity verification, and user service functions into a digitally coordinated service system. Sponsorship conversion restructuring refers to improvements in the precision, continuity, and measurability of sponsor activation under digital operation. Data monetization restructuring captures the extent to which event-related data are converted into commercial products, services, or revenue-generating capabilities. These dimension-specific indicators help unpack the broader restructuring outcome and reveal which parts of the value chain are more responsive to digital upgrading.

Several control variables are included to reduce omitted-variable bias. Event size is controlled because larger events usually have more resources, stronger organizational capacity, and broader market influence, which may independently affect value chain structure. Event level is included to distinguish between top-tier events and lower-tier events with different degrees of marketization and media visibility. Regional market environment is controlled because digital infrastructure, business ecology, and local consumption conditions differ across regions. Audience base is considered because events with a larger and more stable audience may find it easier to build digital communities and monetize user engagement. Sponsorship resource endowment is also controlled because pre-existing commercial capability may shape both the adoption of digital tools and the restructuring of commercial activities. Together, these controls help isolate the net relationship between digital upgrading and value chain restructuring. Table 1 presents the definitions and measurement approaches for the main variables used in the study.

**Table 1:** Definition and measurement of key variables.

Variable type	Variable	Definition	Measurement approach
Explanatory variable	Digital upgrading level	The extent to which digital technologies are embedded in event operations and commercialization activities	Composite index based on content digitization, platform distribution, digital ticketing, sponsor activation tools, and data monetization systems
Outcome variable	Value chain restructuring degree	The extent of structural change in coordination patterns, actor roles, and value allocation within the sport event industry chain	Composite measure based on network density, node centrality shifts, collaboration intensity, and variation in value capture across segments
Structural dimension	Content production restructuring	The extent to which content creation, editing, and dissemination become digitally coordinated and modularized	Indicators such as digital production share, multi-platform output, real-time data integration, and content reuse intensity
	Marketing and distribution restructuring	The extent to which audience reach and promotion rely on platform-based and data-driven mechanisms	Indicators such as platform traffic share, precision marketing intensity, distribution channel integration, and user conversion rate
	Ticketing service restructuring	The extent to which ticketing and audience services are integrated through digital systems	Indicators such as online ticketing ratio, dynamic pricing usage, digital identity verification, membership linkage, and service integration level
	Sponsorship conversion restructuring	The extent to which sponsorship value is activated, tracked, and converted through digital operations	Indicators such as sponsor exposure precision, interactive activation frequency, conversion monitoring capability, and sponsor retention performance
	Data monetization restructuring	The extent to which event and user data are transformed into monetizable products or service capabilities	Indicators such as number of data-based products, data service revenue share, user profiling capability, and commercial use of behavioral data
Control variable	Event size	The scale of the event in terms of participation, viewership, or organizational resources	Attendance, audience size, number of participants, operating budget, or equivalent scale indicators
	Event level	The competitive and commercial ranking of the event	Categorical classification based on event tier, league status, or official recognition
	Regional market environment	The external market conditions surrounding the event	Regional digital infrastructure, income level, business environment, or local market development indicators
	Audience base	The breadth and stability of the event's user foundation	Registered users, repeat audience ratio, fan community size, or social media followers
	Sponsorship resource endowment	The pre-existing strength of sponsor-related commercial resources	Number of sponsors, sponsorship revenue base, sponsor category diversity, and continuity of sponsor relationships

### 2.4 Reconstruction path identification

The qualitative stage is used to identify how digital upgrading reshapes the structure of the sport event value chain. Based on case materials, policy documents, platform records, interviews, and secondary reports, the study adopts an open coding strategy to extract recurring patterns of organizational adjustment, functional redistribution, and commercial change. Coding focuses on changes in actor roles, interface arrangements, coordination mechanisms, and revenue sources across key segments of the value chain. Through iterative comparison within and across cases, conceptually similar observations are grouped into higher-order categories, which allows the analysis to move from dispersed empirical phenomena to comparable restructuring patterns.

Organizational boundary change is identified by examining who controls key activities, who provides specialized functions, and how coordination is governed after digital upgrading. Four forms are emphasized. Internalization refers to the reintegration of strategically important digital functions into the focal organization. Outsourcing reflects the transfer of standardized or non-core tasks to external providers. Platform coordination captures the increasing dependence on digital platforms for traffic allocation, user access, and transaction matching. Ecosystem embedding refers to more stable and multi-sided collaboration among organizers, platforms, sponsors, data firms, and service partners. These boundary changes are treated as observable signals of how digital technologies alter the governance structure of the industry chain.

The reconfiguration of value activities is assessed from three aspects, namely the redistribution of tasks, the strengthening of inter-actor coordination, and the change in revenue allocation. Task redistribution is reflected in shifts in the location of content production, marketing execution, ticketing management, sponsor activation, and data operations. Coordination strengthening is reflected in tighter information sharing, more integrated service processes, and more frequent interaction across organizational interfaces. Revenue allocation change is reflected in the movement of value capture toward data-intensive, platform-mediated, and service-integrated segments. These dimensions help reveal whether digital upgrading merely improves isolated activities or leads to deeper structural adjustment in the value chain.

On the basis of cross-case comparison, the study extracts several recurring restructuring paths and identifies the conditions under which they emerge. Path extraction follows the logic of configuration comparison, linking digital inputs, boundary changes, activity reallocation, and restructuring outcomes. Trigger conditions are identified by observing whether specific combinations of platform capability, data accumulation, audience scale, commercialization maturity, and governance flexibility are associated with stronger restructuring. The aim is not to produce an exhaustive typology, but to clarify the most representative restructuring trajectories and the practical conditions that make them more likely.

### 2.5 Network and value-flow quantification

#### 2.5.1 Industry network construction

The industry network was constructed based on multi-source evidence, including official event documents, partnership announcements, annual reports, sponsorship materials, platform operation records, and interview verification. Actors were identified as organizational entities involved in event-related value activities, and relationships were coded according to documented collaboration, transaction, and digital integration activities. Both binary and weighted networks were constructed to capture the existence and intensity of inter-organizational relationships. Detailed procedures for actor identification, tie coding, data sources, reliability assessment, and network matrix generation are provided in Supplementary Information.

To quantify structural change, the study constructs an industry relationship network for each event case. Nodes represent key actors in the value chain, including event organizers, media platforms, ticketing firms, sponsors, data service providers, and commercial partners. Edges represent observable transactional or collaborative ties, such as content distribution, ticketing integration, data exchange, sponsorship activation, and revenue-sharing arrangements. The network is represented by an adjacency matrix $A=(a_{ij})$, where $a_{ij}=1$ if actor *i* maintains a valid relationship with actor *j*, and $a_{ij}=0$ otherwise. When tie intensity can be observed, a weighted matrix $W=(w_{ij})$ is used, where $w_{ij}$ denotes the strength of interaction, transaction volume, or collaboration frequency between nodes *i* and *j*.

#### 2.5.2 Structural network indicators

The network structure is evaluated through density, centrality, structural holes, and collaborative embeddedness. Network density is used to capture the overall closeness of the industry chain and is calculated as

$$D=\frac{L}{N(N-1)}$$

where *L* is the number of observed ties and *N* is the number of nodes. Degree centrality is used to reflect the relative prominence of actor *i* in the network and is defined as

$$C_{D}(i)=\frac{\sum_{j}a_{ij}}{N-1}$$

Betweenness centrality is used to assess brokerage capacity and is measured as

$$C_{B}(i)=\underset{\;j<k}{\sum }\;\frac{g_{\;jk}(i)}{g_{\;jk}}$$

where $g_{\;jk}$ is the number of shortest paths between nodes *j* and *k*, and $g_{\;jk}(i)$ is the number of those paths passing through node *i*. Structural hole advantage is captured by Burt's constraint index

$$Constraint_{i}=\underset{j}{\sum }\;{\left(\;p_{ij}+\underset{q}{\sum }\;p_{iq}p_{qj}\right)}^{2}$$

where a lower value indicates stronger brokerage opportunities. Collaborative embeddedness is measured by the weighted average strength of repeated and multiplex ties among core actors, which reflects whether the network is evolving toward more stable platform-based coordination rather than fragmented linear exchange.

#### 2.5.3 Value-flow measurement

Beyond network form, the study measures how value flows shift across the industry chain. Let $R_{i}$ denote the revenue captured by node *i*, and let *V* denote the total observable value generated within the focal event chain. The value capture share of node *i* is defined as

$$S_{i}=\frac{R_{i}}{V}$$

Changes in $S_{i}$ over time indicate whether digital upgrading reallocates value toward particular segments. To describe value transfer between segments, the study constructs a value-flow matrix $F=(\;f_{ij})$, where $f_{ij}$ denotes the value, traffic, or monetizable user flow moving from node *i* to node *j*. Net value inflow for node *i* is measured as

$$NV_{i}=\underset{j}{\sum }\;f_{\;ji}-\underset{j}{\sum }\;f_{ij}$$

A positive $NV_{i}$ indicates that the node becomes a stronger value absorber after digital upgrading. Where data permit, a simplified input-output style coefficient is used to capture the dependence of downstream value creation on upstream digital inputs

$$b_{ij}=\frac{\;f_{ij}}{x_{j}}$$

where $x_{j}$ is the total output of node *j*. This helps show whether digital infrastructures increasingly drive revenue generation in downstream marketing, ticketing, sponsorship, and data services.

#### 2.5.4 Reconstruction intensity and phase classification

The intensity of value chain restructuring is measured through a composite index that combines changes in network structure and value allocation. Let ΔD, ΔC, ΔS, and ΔE denote standardized changes in density, centrality distribution, value capture share, and collaborative embeddedness. The restructuring intensity index is defined as

RI=αΔD+βΔC+γΔS+δΔE

where α, β, γ, and δ are weights determined by theoretical importance or empirical normalization. Based on the distribution of RI, the study classifies restructuring into three stages, namely early adjustment, intermediate coordination, and deep restructuring. This classification is used to compare whether different events exhibit only partial digital adaptation or more comprehensive reorganization of the value chain.

### 2.6 Causal identification strategy

To identify whether digital upgrading is associated with structural performance changes, the study applies an event study around major digital upgrading milestones, such as the launch of an integrated digital ticketing system, a platform partnership, or a data-driven sponsorship program. The baseline specification is

$$Y_{it}=\alpha +\underset{k\neq -1}{\sum }\;\beta_{k}D_{i,t+k}+\mu_{i}+\lambda_{t}+\varepsilon_{it}$$

where $Y_{it}$ denotes the performance outcome of event *i* in period *t*, $D_{i,t+k}$ is a set of event-time dummies, $\mu_{i}$ captures event fixed effects, and $\lambda_{t}$ captures time fixed effects. The coefficients $\beta_{k}$ trace the dynamic change in outcomes before and after the digital upgrading event. This design is used to assess whether restructuring-related indicators and performance variables display significant shifts around the intervention window. To further test the effect of digital upgrading, the study employs a difference in differences model comparing events with substantive digital upgrading to comparable events without such changes in the same period. The benchmark model is

$$Y_{it}=\alpha +\beta (Treat_{i}\times Post_{t})+\theta X_{it}+\mu_{i}+\lambda_{t}+\varepsilon_{it}$$

where $Treat_{i}$ indicates whether event *i* belongs to the treated group, $Post_{t}$ indicates the post-upgrading period, and $X_{it}$ is a vector of control variables. The coefficient β captures the average treatment effect of digital upgrading on key outcomes such as ticketing efficiency, sponsorship conversion, user engagement, and data-related revenue performance. This approach allows the analysis to move beyond descriptive comparison and assess whether digital upgrading is linked to structural changes in measurable outcomes.

Several robustness checks are conducted to improve identification credibility. Parallel trend tests are used to examine whether treated and control events followed similar pre-treatment trends. Placebo tests are implemented by assigning false intervention dates or pseudo-treated groups to verify that the estimated effects are not driven by unrelated shocks. Alternative variable specifications are used to test whether results hold under different measures of digital upgrading and restructuring. Heterogeneity analysis is then conducted by splitting the sample according to platform capability, data asset base, event type, and commercialization maturity, in order to determine whether the impact of digital upgrading varies across organizational and market conditions. Together, these tests help assess the stability and boundary conditions of the estimated effects.

To improve the validity of causal identification, this study defines digital upgrading treatment as a substantial organizational-level digital intervention that changes value activities or coordination mechanisms, rather than isolated adoption of digital tools. Eligible treatment events include major digital initiatives such as integrated digital ticketing systems, platform-based ecosystem partnerships, and data-driven engagement systems that directly affect operational processes, user interaction, or commercial conversion. These interventions share a common feature that digital technologies are embedded into core value activities and generate observable changes in organizational coordination, while differences in specific applications are considered through heterogeneity analysis.

Treatment assignment was determined based on documented evidence of digital upgrading events, including official announcements, annual reports, partnership disclosures, and operational records. Events were classified into treatment and comparison groups according to whether they implemented substantive digital upgrading during the observation period. To reduce selection concerns, the study applies event fixed effects, time fixed effects, and control variables including event size, event level, regional market environment, audience base, and sponsorship resource endowment. In addition, robustness tests using alternative treatment definitions and placebo interventions are conducted to examine whether the estimated effects are sensitive to model specification.

### 2.7 Qualitative comparative analysis of reconstruction paths

The qualitative analysis followed a structured three-stage coding process to identify the mechanisms through which digital upgrading reshapes the sport event value chain. In the first stage, open coding was conducted by examining case materials line by line, including interview transcripts, event operation reports, platform announcements, sponsorship documents, and industry reports. Initial codes were generated based on observable changes in actor roles, operational processes, technology adoption patterns, and value creation activities. Examples of initial codes included digital content co-production, algorithm-based audience targeting, integrated ticketing systems, sponsor data tracking, and user data commercialization.

In the second stage, related initial codes were compared, merged, and organized into intermediate themes through constant comparison across cases. Codes describing changes in internal capabilities, control over key activities, and strategic retention of digital functions were grouped into the theme of boundary internalization. Codes related to platform dependence, external partnerships, and multi-actor coordination were consolidated into the theme of ecosystem-based collaboration. Similarly, observations concerning task modularization, digital interfaces, and real-time information exchange were integrated into value activity reconfiguration themes.

In the third stage, higher-order categories were developed by linking intermediate themes with the theoretical framework of digital upgrading, organizational boundaries, and value chain restructuring. The final categories included boundary contraction and capability consolidation, boundary expansion and platform ecosystem coordination, modular value activity reconfiguration, and data-driven value capture extension. These categories were then compared across cases to identify common restructuring paths and their triggering conditions.

To enhance reliability, the coding process involved two independent researchers with backgrounds in sport management and digital transformation research. The first researcher conducted the initial coding, while the second researcher independently reviewed the coding results and assessed category consistency. Disagreements were discussed through iterative comparison until consensus was reached. The intercoder agreement reached 0.86, indicating substantial consistency between coders. The final coding framework was established after three rounds of refinement and was used for subsequent cross-case analysis.

## 3 Result

### 3.1 Overall pattern of digital upgrading and value-chain change

Table 2 reports the descriptive statistics for the core variables. The sample covers 36 sport events observed over 2019–2024, yielding 216 event-year observations. The mean value of digital upgrading is 0.581, with a standard deviation of 0.174, indicating clear variation in the depth of digital transformation across events. Among the structural dimensions, marketing and distribution restructuring shows the highest mean value, followed by ticketing service restructuring, suggesting that digital change has been more visible in audience reach, channel integration, and transaction-related services. Data monetization restructuring records the lowest mean value and the largest dispersion, which implies that although data-driven commercialization has emerged in part of the sample, it remains uneven and concentrated in events with stronger platform resources and user data accumulation. As shown in Table 3, the mean value of overall value chain restructuring is 0.547, indicating that digital upgrading has already produced observable structural effects, but the depth of change is still differentiated across event types and commercialization contexts.

**Table 2:** Coding process from initial concepts to higher-order categories.

Empirical observations	Initial codes	Intermediate themes	Higher-order categories
Event organizations establish internal digital production teams and retain control over core content assets	Internal digital capability building; control retention; content ownership	Internal capability consolidation	Boundary contraction and core capability recovery
Events cooperate with platforms for traffic distribution, user access, and commercial conversion	Platform dependence; traffic coordination; external digital partnership	Ecosystem-based collaboration	Boundary expansion and platform ecosystem coordination
Ticketing, membership, and service systems become integrated through shared digital interfaces	Service integration; interface standardization; process modularization	Digital service coordination	Value activity modularization and real-time collaboration
Sponsors use user data and interaction analytics to evaluate commercial returns	Data-driven sponsorship activation; user profiling; conversion tracking	Data-enabled commercialization	Data-based value capture extension
Event data are transformed into products, services, or personalized user experiences	Data asset development; behavioral analytics; digital monetization	Data value creation	Deep value chain restructuring

**Table 3:** Descriptive statistics of main variables.

Variable	N	Mean	SD	Min	Max
Digital upgrading level	216	0.581	0.174	0.21	0.91
Value chain restructuring degree	216	0.547	0.161	0.19	0.88
Content production restructuring	216	0.562	0.149	0.22	0.86
Marketing and distribution restructuring	216	0.614	0.158	0.25	0.9
Ticketing service restructuring	216	0.598	0.166	0.18	0.91
Sponsorship conversion restructuring	216	0.521	0.173	0.14	0.86
Data monetization restructuring	216	0.441	0.192	0.08	0.83
Network density	216	0.346	0.097	0.12	0.59
Collaborative embeddedness	216	0.487	0.138	0.15	0.79
Event size	216	10.842	1.126	8.214	13.406
Audience base	216	12.117	1.304	8.976	14.885

The descriptive results suggest a staged pattern of digital upgrading. At the early stage, digital adoption was concentrated in online communication, basic ticketing systems, and fragmented platform presence, with limited penetration into core operational and commercial functions. At the intermediate stage, digital tools became more integrated into content distribution, audience management, and sponsor activation, and the organizational role of platforms began to expand. At the more advanced stage, digital upgrading extended beyond operational support and became embedded in value creation itself, especially through integrated data systems, multi-platform coordination, and more continuous user conversion mechanisms. In the sample, leading professional events are more frequently located in the advanced stage, while mass participation events tend to cluster in the intermediate stage, and comprehensive events show stronger internal variation due to more complex governance arrangements and heterogeneous commercialization structures.

The initial changes in the division of labor and collaboration across value activities are also evident. Content production has become less dependent on single-channel media logic and more oriented toward modular production and cross-platform circulation. Marketing and distribution have shifted from one-way exposure to data-driven reach and dynamic audience interaction, which has strengthened the role of platforms as coordinating nodes. Ticketing is no longer an isolated transaction function, but increasingly connected with membership management, identity verification, and service delivery. Sponsorship activation shows a gradual move from static exposure to trackable and scenario-based conversion, though the degree of improvement varies across events. Data monetization remains the least mature segment, but its growth is closely associated with higher levels of collaborative embeddedness and stronger centrality of digital intermediaries. Taken together, these descriptive patterns indicate that digital upgrading is accompanied by a gradual transition from segmented operations toward a more connected and platform-mediated value chain structure.

### 3.2 Cross-case comparison of reconstruction paths

Cross-case comparison shows that digital upgrading does not produce a single restructuring trajectory. Instead, four relatively stable paths emerge, each with a different leading segment and a different pattern of boundary adjustment, coordination change, and value capture. Figure 1 visualizes these differences. The content-led path is characterized by the strongest adjustment in content production, with a segment score of 0.74, clearly higher than marketing distribution at 0.58 and ticketing service at 0.49. This pattern suggests that digital upgrading initially operates through the reorganization of media production, multi-platform content circulation, and real-time data enrichment. As shown in Figure 1b, the centrality of media-related actors rises from 0.41 to 0.57, while the organizer also strengthens its coordinating role from 0.62 to 0.68. Figure 1c shows that organizers capture 31 percent of value after upgrading, the highest share among the four paths, indicating that content control remains closely tied to the focal event organization. The restructuring intensity of this path is 0.53, which is lower than the platform-driven and data-asset extension paths, suggesting that content-led restructuring improves value creation but does not always produce the deepest reordering of the entire chain.

**Figure 1:**
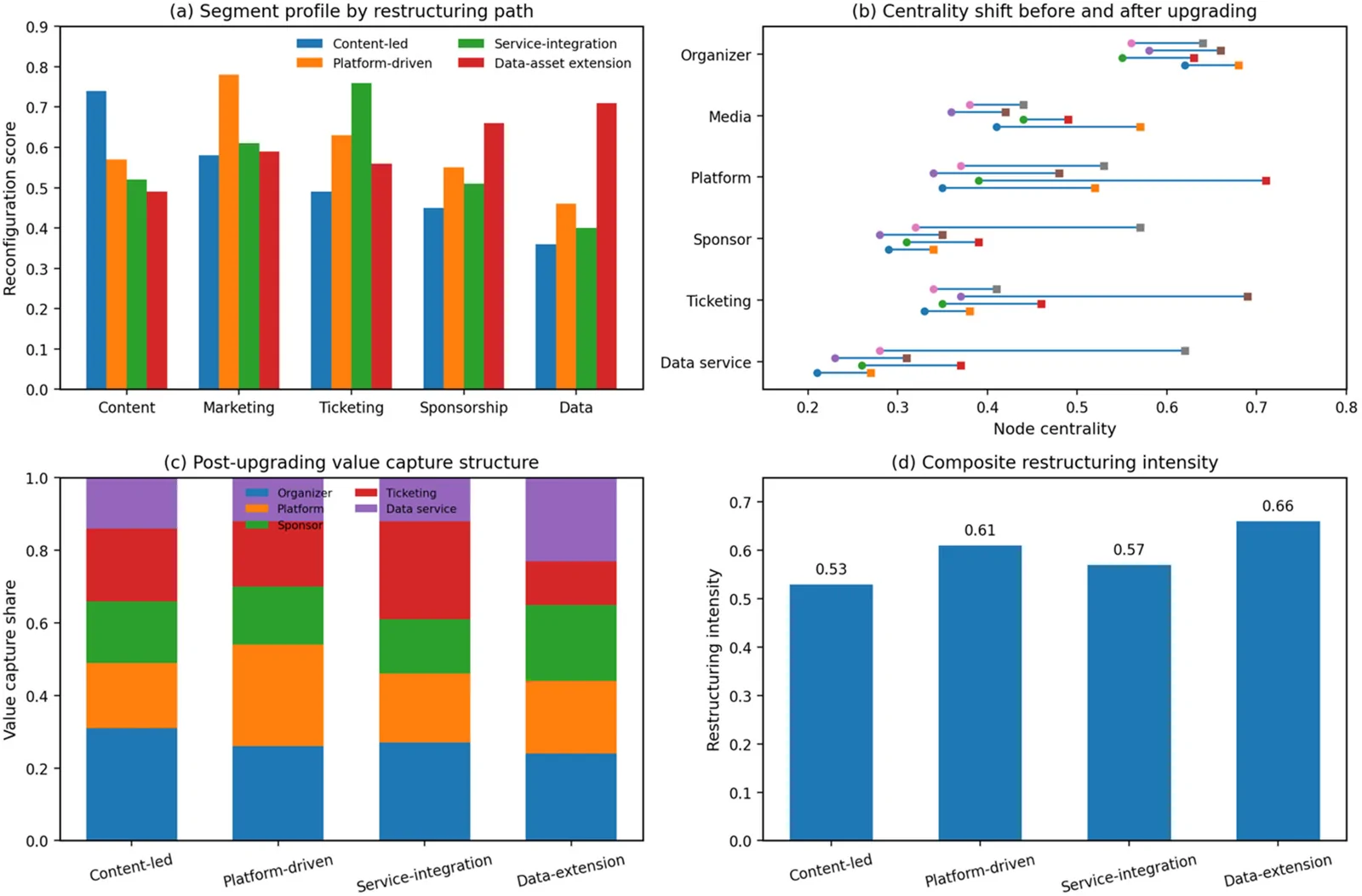
Cross-case comparison of restructuring paths in the sport event industry. **(a)** shows the reconfiguration scores of five value segments across four restructuring paths. **(b)** reports the changes in node centrality before and after digital upgrading. **(c)** presents the post-upgrading value capture shares of key actors. **(d)** summarizes the composite restructuring intensity index.

The platform-driven path shows the clearest change in marketing distribution, with a score of 0.78, the highest single dimension among all four paths. This result indicates that platform-based traffic allocation, precision targeting, and algorithmic distribution have become the primary drivers of restructuring in this group of events. The centrality shift in Figure 1b is particularly pronounced for the platform node, which rises from 0.39 to 0.71, exceeding all other actors. Figure 1c further shows that platforms account for 28 percent of post-upgrading value capture, higher than organizers at 26 percent. This confirms that digital upgrading in these cases tends to re-center the value chain around distribution platforms rather than traditional event operators. The restructuring intensity reaches 0.61, indicating a relatively deep transition from segmented coordination to platform-centered collaboration.

The service-integration path is most evident in ticketing and user service functions. Its ticketing service restructuring score reaches 0.76, while the other segment scores remain between 0.40 and 0.61. This pattern reflects the growing integration of ticketing, membership, identity verification, and event service processes within a unified digital system. Figure 1b shows that the centrality of ticketing-related actors increases sharply from 0.37 to 0.69, a larger gain than in any other path except the platform node in the platform-driven group. Figure 1c also indicates that ticketing-related functions capture 27 percent of value, equal to the organizer share and above the platform share. The restructuring intensity is 0.57, placing this path in the middle range. The results suggest that service integration improves operational coherence and user linkage, but its effect on broader value reallocation remains more moderate than in cases where distribution or data assets dominate restructuring.

Although the four restructuring paths capture the dominant patterns identified across cases, cross-case analysis also reveals that digital upgrading does not always lead to successful value chain restructuring. Several cases exhibited hybrid configurations in which digital capabilities improved specific operational functions but failed to generate broader organizational or commercial transformation. For example, some mass participation events successfully introduced digital registration and mobile service systems, yet limited data accumulation and weak commercialization capabilities prevented further development toward data-driven value creation. In these cases, digital upgrading remained concentrated at the service efficiency level rather than extending to deeper value chain reconfiguration.

Contradictory observations were also identified in platform-oriented restructuring. While platform partnerships improved audience reach and traffic acquisition, excessive dependence on external platforms created governance tensions related to data ownership, revenue distribution, and strategic control. Some organizers experienced increased operational efficiency but reduced control over user relationships and commercial data, indicating that platform integration may generate both coordination benefits and dependency risks.

The comparison further shows that digital upgrading outcomes depend on the alignment between technological adoption and organizational capability. Events with fragmented internal processes, limited data governance capacity, or unclear commercialization strategies often encountered difficulties in transforming digital resources into sustainable value creation. These cases suggest that digital upgrading should be understood as a conditional restructuring process rather than an automatic pathway toward higher-value configurations.

### 3.3 Organizational boundary change and value-activity reconfiguration

The results show that digital upgrading is associated with selective boundary contraction rather than simple organizational expansion. In the content-led path, the internalization index reaches 0.63, the highest among the four paths, indicating a clear return of strategically important functions to the focal organization. This pattern is concentrated in content planning, production control, and user-facing data operations, where direct control improves coordination efficiency and protects key assets. As shown in Figure 2a, the corresponding platform dependence of this group remains relatively limited at 0.38, suggesting that digital upgrading can strengthen internal control when the event organization retains strong content and brand capabilities. This pattern supports the view that boundary contraction is most likely when digital tools enhance the manageability of core functions rather than replacing them with external intermediaries.

**Figure 2:**
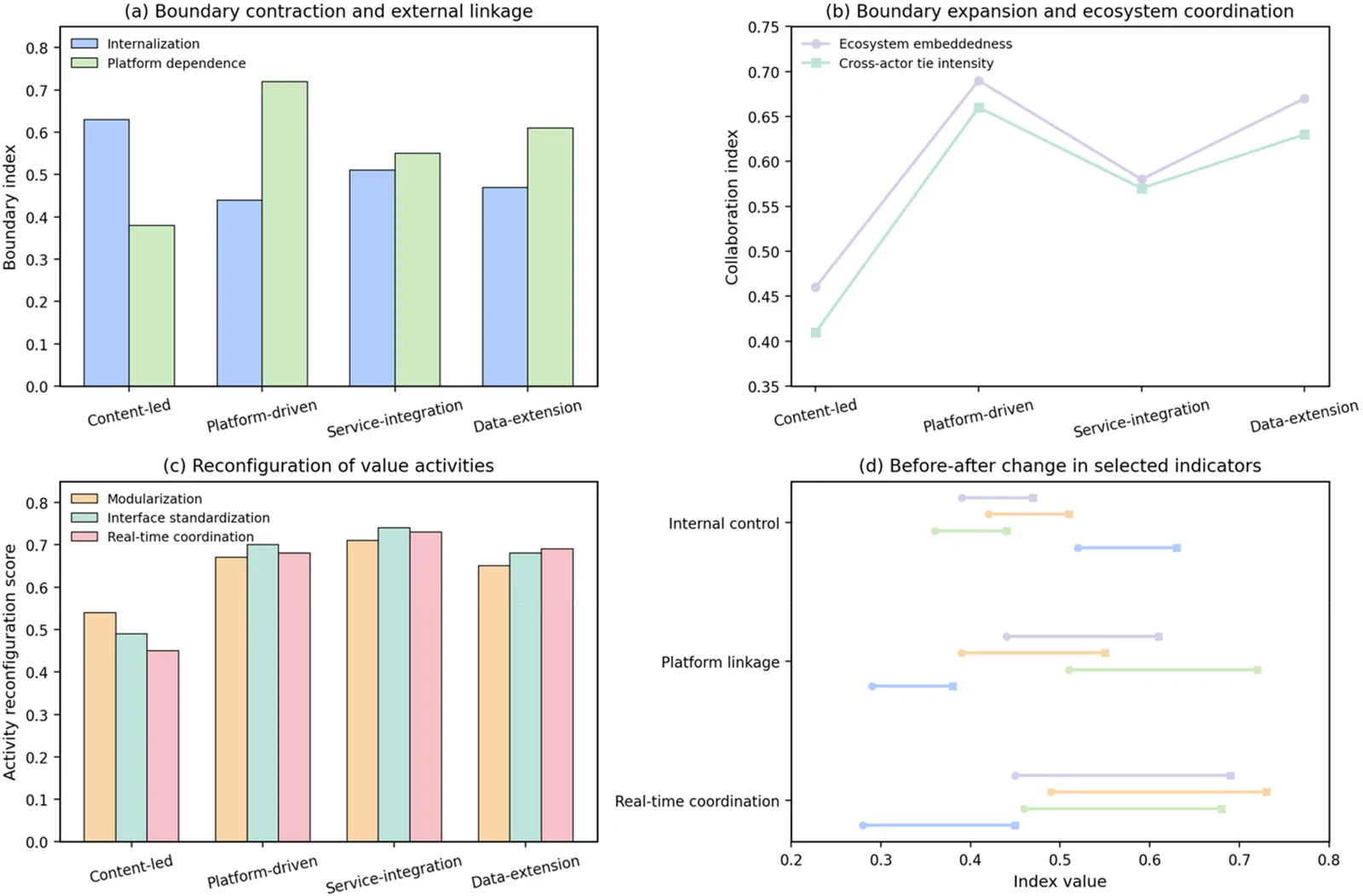
Organizational boundary change and value-activity reconfiguration across four restructuring paths. **(a)** compares internalization and platform dependence, showing how digital upgrading reshapes the balance between internal control and external linkage. **(b)** reports ecosystem embeddedness and cross-actor tie intensity, indicating the degree of outward boundary expansion and collaborative coordination. **(c)** presents the reconfiguration scores of modularization, interface standardization, and real-time coordination across value activities. **(d)** compares selected indicators before and after digital upgrading.

A different pattern appears in the platform-driven and data-extension paths, where digital upgrading leads to outward boundary expansion and closer ecosystem coordination. In the platform-driven path, platform dependence rises to 0.72, while ecosystem embeddedness reaches 0.69, both higher than in the other groups. In the data-extension path, the corresponding values are 0.61 and 0.67. These results suggest that digital upgrading increasingly relies on stable collaboration with platforms, sponsors, and data service firms, especially in activities related to distribution, conversion, and monetization. Figure 2b further shows that cross-actor tie intensity reaches 0.66 in the platform-driven path and 0.63 in the data-extension path, confirming that deeper restructuring is associated with a denser external collaboration network. In these cases, value creation depends less on isolated organizational control and more on coordinated access to traffic, data, and commercial interfaces.

The reconfiguration of value activities is reflected not only in who performs key tasks, but also in how these tasks are organized and connected. Figure 2c shows that the service-integration path records the highest scores for modularization at 0.71, interface standardization at 0.74, and real-time coordination at 0.73. This indicates that ticketing, membership, identity verification, and on-site service functions are increasingly organized as interoperable modules linked through standardized digital interfaces. The platform-driven path also shows high scores in this dimension, with interface standardization at 0.70 and real-time coordination at 0.68, suggesting that platform-based restructuring relies on stable digital interfaces across actors. By contrast, the content-led path remains more moderate, with real-time coordination at 0.45, which implies that stronger control over core capabilities does not necessarily produce the highest level of process modularization. Overall, the evidence indicates that digital upgrading promotes a transition from fragmented task execution to modular, interface-based, and real-time collaborative operation.

The before-and-after comparison in Figure 2d further clarifies the joint evolution of boundaries and activities. Internal control rises from 0.52 to 0.63 in the content-led path, while platform linkage increases more sharply in the platform-driven path, from 0.51 to 0.72. Real-time coordination shows the largest improvement in the service-integration path, from 0.49 to 0.73, and remains high in the data-extension path, increasing from 0.45 to 0.69. These patterns indicate that organizational boundary change and value-activity reconfiguration are closely aligned but not uniform across cases. Some events strengthen internal control over core capabilities, while others expand outward and rely on ecosystem coordination. Across all paths, however, digital upgrading is consistently associated with more structured interfaces, tighter collaboration, and a clearer redistribution of value-generating tasks across the event industry chain.

### 3.4 Network structure and value-flow change

The network analysis shows that digital upgrading is associated with increased connectivity among actors in the sport event value chain. As network density increases after digital upgrading, more direct relationships among organizations are observed. This structural change suggests a potential increase in interaction opportunities and relational connectivity; however, higher density does not necessarily indicate effective coordination or improved governance outcomes. Increased connectivity may also reflect greater dependence among actors, expanded transaction frequency, or the emergence of more complex inter-organizational relationships. Therefore, network density is interpreted here as evidence of structural association rather than direct evidence of coordination effectiveness. As shown in Figure 3a, network density rises in all four restructuring paths, from 0.29 to 0.35 in the content-led path, from 0.31 to 0.41 in the platform-driven path, from 0.30 to 0.37 in the service-integration path, and from 0.32 to 0.43 in the data-extension path. This pattern is consistent with the descriptive finding that digital upgrading weakens segmented exchange and strengthens more connected coordination. The increase is most pronounced in the platform-driven and data-extension paths, suggesting that deeper digital upgrading is more likely to generate denser industrial linkages. Figure 3b further shows that post-upgrading centrality is no longer concentrated solely in the organizer. The organizer remains important, with centrality ranging from 0.63 to 0.68 across paths, but platform, ticketing, sponsor, and data service actors all gain relevance in specific contexts, indicating a more differentiated and functionally distributed network structure.

**Figure 3:**
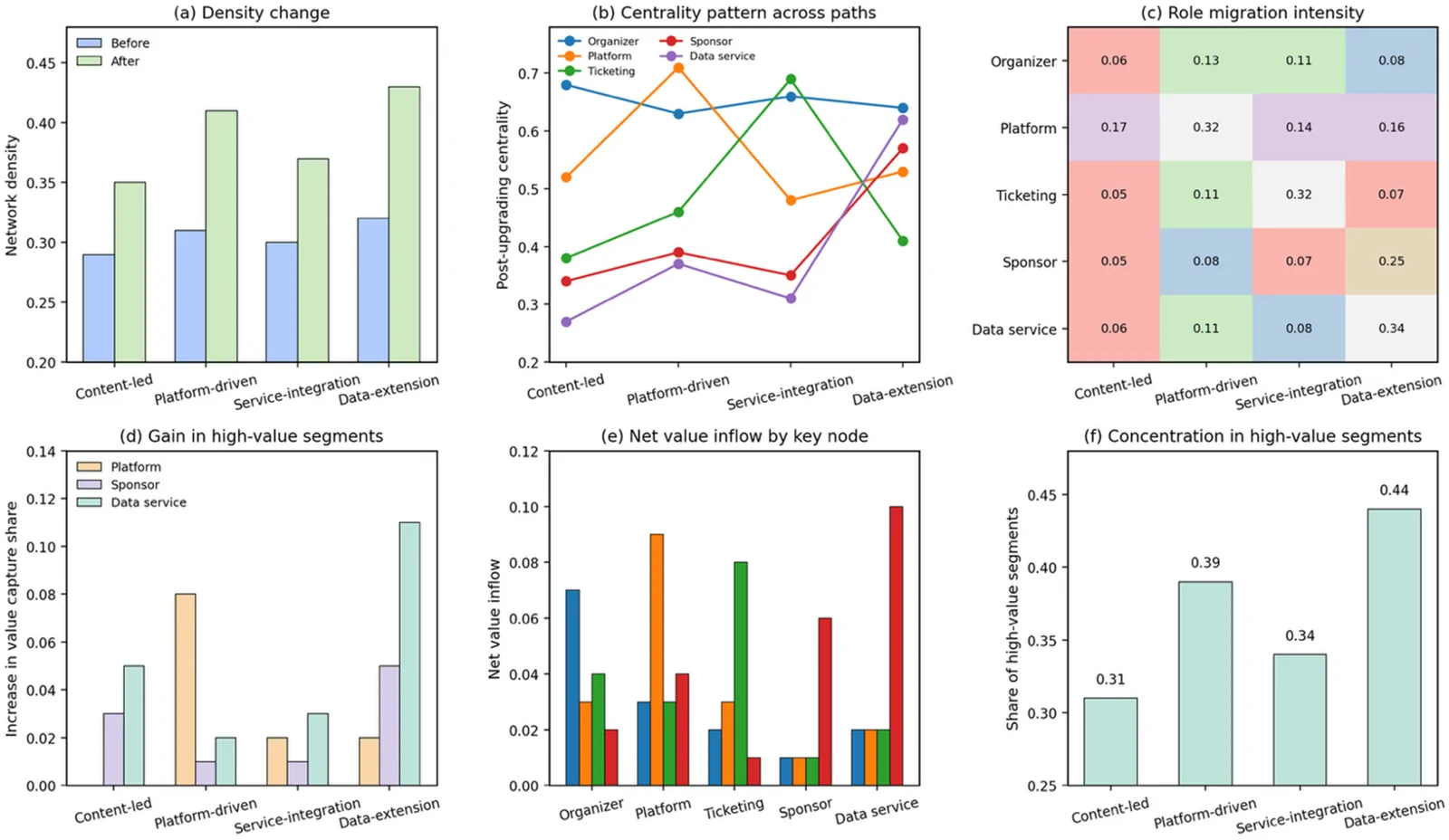
Network structure and value-flow change across four restructuring paths. **(a)** reports the change in network density before and after digital upgrading. **(b)** presents post-upgrading centrality patterns of key actors. **(c)** shows the intensity of role migration measured by centrality gains. **(d)** compares the increase in value capture share across high-value segments. **(e)** reports net value inflow by key node. **(f)** summarizes the concentration of value in high-value segments after digital upgrading.

Role migration is not uniform across actor types. Figure 3c shows that the platform node records the largest centrality gain in the platform-driven path, increasing by 0.32, while the ticketing node gains 0.32 in the service-integration path. In the data-extension path, the most prominent gains occur in the data service node at 0.34 and the sponsor node at 0.25. By contrast, the organizer shows smaller but stable gains, ranging from 0.06 to 0.13 across paths. These results suggest that digital upgrading does not displace the organizer, but it reduces the exclusivity of its coordinating role by elevating specialized actors in segments with stronger digital intermediation. The network therefore evolves from organizer-centered coordination toward a more polycentric structure in which control and brokerage functions are increasingly distributed across platforms, service providers, and commercialization partners.

The observed changes in value-flow distribution indicate that digital upgrading is associated with shifts in the relative importance of different value-generating activities. The increased contribution of platform, sponsorship, or data-related segments suggests that digitally enabled activities become more prominent in commercial value creation. However, these changes should not be interpreted as direct evidence of complete value chain restructuring, because value redistribution may also result from market expansion, changes in consumer behavior, or broader industry trends. The findings provide empirical evidence of changing value patterns that are consistent with restructuring processes. Figure 3d shows that the increase in value capture is concentrated in high-value segments. In the platform-driven path, the platform share rises by 0.08, while sponsor and data service gains remain limited. In the data-extension path, the sponsor share increases by 0.05 and the data service share by 0.11, both higher than in the other groups. Figure 3e confirms this pattern from the perspective of net value inflow. In the content-led path, the organizer records the largest inflow at 0.07. In the platform-driven path, the highest inflow appears in the platform node at 0.09. In the service-integration path, the ticketing node becomes the main value absorber with an inflow of 0.08. In the data-extension path, the largest inflows shift to data service at 0.10 and sponsor at 0.06. Figure 3f further shows that the combined value share of high-value segments, including platform, sponsor, and data service actors, reaches 0.44 in the data-extension path, compared with 0.39 in the platform-driven path, 0.34 in the service-integration path, and 0.31 in the content-led path. These results indicate that digital upgrading not only increases network connectivity, but also moves the center of value capture toward digitally mediated and commercially scalable segments.

### 3.5 Causal test results

To strengthen causal identification, the event-study design is anchored to publicly observable digital upgrading milestones in major sport events. Representative cases include the launch of the official Paris 2024 Tickets app in May 2024, which enabled ticket download, transfer, and resale, and the deployment of UEFA EURO 2024 mobile ticketing, where digital mobile tickets became the default access mechanism. The treated sample also includes events with documented platform and data integration initiatives, such as Formula 1's fan-engagement partnership with Salesforce and IRONMAN's digital training and app-integration partnership with ROUVY. These cases provide clear intervention windows for identifying the short-term and lagged effects of digital upgrading on value-chain restructuring.

The dynamic estimates show no significant pre-trend and a clear post-event improvement in restructuring-related outcomes. As reported in Table 4 and visualized in Figure 4a, the coefficients in periods t−3 and t−2 are small and statistically insignificant, which supports the parallel-trend assumption. The effect becomes positive in the event period and increases further in the subsequent two periods. The coefficient rises from 0.041 in period *t* to 0.063 in period t+1 and 0.074 in period t+2, before stabilizing at 0.069 in period t+3. This pattern suggests that the impact of digital upgrading is not purely instantaneous. It accumulates as new digital interfaces are absorbed into organizational routines, external linkages become denser, and high-value activities gradually shift toward platform-enabled and data-intensive segments.

**Table 4:** Event-study estimates of digital upgrading effects.

Event time	Coefficient	SE	*t* value	*p* value
t−3t	−0.012	0.01	−1.2	0.231
t−2t-	−0.006	0.009	−0.67	0.505
t	0.041	0.015	2.73	0.007
t + 1	0.063	0.018	3.5	0.001
t + 2	0.074	0.02	3.7	0
t + 3	0.069	0.021	3.29	0.001

**Figure 4:**
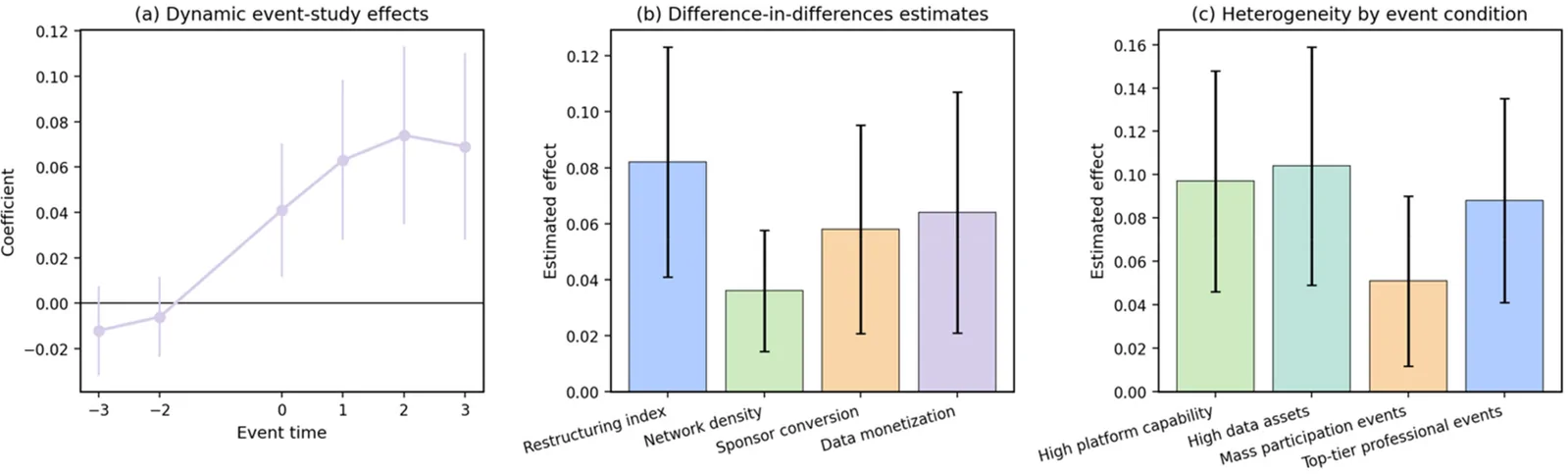
Causal test results of digital upgrading in the sport event industry. **(a)** reports the dynamic event-study coefficients around major digital upgrading events and shows that the treatment effect emerges after the intervention rather than before it. **(b)** presents the DID estimates for the restructuring index, network density, sponsorship conversion, and data monetization. **(c)** reports heterogeneous treatment effects across different event conditions. The overall pattern indicates that digital upgrading has a significant and persistent effect on value-chain restructuring, with stronger impacts in events that possess greater platform capability and richer data assets.

The difference-in-differences results provide further evidence that digital upgrading leads to measurable structural change. Table 5 shows that the treatment effect on the overall restructuring index is 0.082 and remains statistically significant at the 1 percent level. The effect on network density is 0.036, indicating that treated events develop tighter inter-organizational ties after upgrading. The coefficient for sponsorship conversion is 0.058, while the effect for data monetization reaches 0.064. These estimates are consistent with the network and value-flow results reported above. Digital upgrading not only improves connectivity, but also shifts value capture toward commercially scalable and digitally mediated segments. Figure 4b presents these estimates graphically and shows that the effects are strongest for the restructuring index and data monetization, followed by sponsorship conversion and network density.

**Table 5:** Difference-in-differences estimates and robustness tests.

Dependent variable	DID coefficient	SE	*p* value	Placebo coefficient	Alternative measure coefficient
Value chain restructuring degree	0.082	0.021	0	0.011	0.079
Network density	0.036	0.011	0.002	0.005	0.034
Sponsorship conversion restructuring	0.058	0.019	0.003	0.008	0.055
Data monetization restructuring	0.064	0.022	0.004	0.01	0.061

The identification tests support the stability of the main findings. The event-study results show no meaningful pre-treatment divergence, and the placebo coefficients in Table 4 remain small and statistically insignificant. Re-estimation with alternative measures produces coefficients close to the benchmark model, which suggests that the results are not driven by a single operationalization of digital upgrading or restructuring. Figure 4c further shows that the treatment effect is heterogeneous across event conditions. The coefficient is strongest in events with high data assets at 0.104 and high platform capability at 0.097, followed by top-tier professional events at 0.088. The effect is weaker, though still positive, in mass participation events at 0.051. This pattern is fully consistent with Hypothesis 3. Digital upgrading generates deeper restructuring where platform coordination is stronger, data resources are richer, and commercialization scenarios are more mature.

To further evaluate the stability of the quantitative findings, additional robustness analyses were conducted using alternative measurements, placebo tests, and sample adjustments. The baseline treatment definition identifies digital upgrading events as organizational-level digital initiatives that directly affect value activities, including integrated digital ticketing systems, platform-based operating partnerships, and data-enabled engagement mechanisms. To reduce concerns that different forms of digital upgrading may represent heterogeneous interventions, alternative specifications were estimated by excluding single-function digital initiatives and retaining only comprehensive digital upgrading events involving at least two value-chain dimensions.

The robustness results remain consistent with the baseline findings. As shown in Table 6, the coefficient of digital upgrading on value chain restructuring remains positive and significant after replacing the restructuring index with alternative network-based and value-flow-based measures. The estimated effect decreases slightly from 0.082 in the baseline model to 0.076 under the alternative specification, but remains statistically significant. This indicates that the relationship between digital upgrading and restructuring is not driven by a single measurement approach.

**Table 6:** Robustness tests of digital upgrading effects.

Specification	Dependent variable	Coefficient	SE	*p* value
Baseline model	Value chain restructuring index	0.082	0.021	0
Alternative measure 1	Network restructuring index	0.076	0.023	0.001
Alternative measure 2	Value-flow restructuring index	0.079	0.024	0.001
Excluding single-function upgrades	Composite restructuring index	0.074	0.025	0.003

Given that digitally advanced events may possess stronger financial resources, larger audiences, and superior managerial capabilities, additional analyses were conducted to examine whether the observed effects are mainly driven by pre-existing advantages. The models were re-estimated after controlling for event scale, audience base, sponsorship resources, event level, and regional market conditions. The results remain stable, suggesting that digital upgrading is associated with restructuring outcomes beyond these observable characteristics. A placebo test was further conducted by randomly assigning pseudo-treatment years to untreated events. The estimated placebo coefficient was 0.009 and statistically insignificant, which indicates that the main effect is unlikely to be generated by random temporal changes or unrelated industry trends. However, because digital upgrading decisions are not fully exogenous, remaining endogeneity concerns cannot be completely excluded. Therefore, the results should be interpreted as strong empirical evidence of the relationship between digital upgrading and value chain restructuring rather than definitive proof of a fully exogenous causal effect.

Further heterogeneity analysis reveals that the effect of digital upgrading varies according to organizational and market conditions. Events with high platform capability show a restructuring coefficient of 0.097, while events with stronger data assets exhibit an effect of 0.104. By contrast, mass participation events show a relatively lower coefficient of 0.051. These results indicate that digital upgrading generates stronger restructuring effects when organizations possess sufficient digital infrastructure, data accumulation, and commercialization capacity. Overall, the robustness and heterogeneity results support the central argument that digital upgrading is associated with deeper value chain restructuring, while also demonstrating that the magnitude of this relationship depends on the organizational resources and ecosystem conditions of sport events.

## 4 Discussion

This study extends research on digital transformation in the sport event industry by shifting the analytical focus from technology adoption at the functional level to structural change at the industry-chain level. Existing studies have largely examined how digital tools improve communication efficiency, fan engagement, or service quality, yet have paid less attention to how digital upgrading reshapes the architecture of value creation across interdependent actors. By showing that digital transformation is associated with differentiated restructuring paths, this study reframes digitalization as an industry-organizing process rather than a set of isolated technological applications. The findings also advance the application of organizational boundary theory in the sport event context. In event industries, boundary change is rarely expressed as a simple movement toward internalization or outsourcing alone. The evidence suggests a more selective pattern in which some functions return to the focal organization, while others become more deeply embedded in platform and ecosystem coordination. This makes the sport event industry a useful setting for understanding how digital technology alters control, interdependence, and governance under conditions of multi-actor collaboration, public visibility, and commercial complexity. In addition, the study contributes to the literature on value chain restructuring and platform-based coordination by identifying the structural consequences of digital upgrading for network density, node centrality, and value capture. The results show that value chain restructuring does not only involve efficiency improvement, but also a redistribution of brokerage roles and revenue opportunities across organizers, platforms, ticketing actors, sponsors, and data service firms. This helps clarify how platform coordination and data-based commercialization become embedded in the reorganization of industry relationships.

The mechanism analysis suggests that digital technology reshapes the value chain through three closely related channels. It changes control by making some key functions more measurable, standardized, and manageable within the focal organization. It changes connection by increasing the frequency and stability of cross-actor interaction through digital interfaces, shared data systems, and platform infrastructures. It changes value capture by shifting revenue opportunities toward segments that control user access, data processing, traffic allocation, and conversion scenarios. In this sense, the effect of digital upgrading is structural because it changes both the locus of coordination and the distribution of commercial returns. The results also indicate that restructuring across value segments is sequential rather than simultaneous. Marketing and distribution, along with ticketing and user service, tend to respond earlier because these functions are directly linked to platform access, transaction efficiency, and audience management. Content production follows when digital circulation and multi-platform adaptation become central to event visibility. Sponsorship conversion and data monetization usually deepen later, once stable user data, interaction scenarios, and commercialization interfaces have been formed. This order helps explain why some events show visible digital adaptation without reaching deep value-chain restructuring.

The triggering conditions are likewise configurational. Stronger platform capability, richer data assets, and more mature commercialization scenarios reinforce one another and make deeper restructuring more likely. By contrast, when digital infrastructure is fragmented, audience data are limited, or governance arrangements remain rigid, digital upgrading may improve selected functions without substantially altering the overall chain structure. The effect of digital technology is therefore conditioned by organizational readiness and market context rather than determined by technology alone. For event organizers, the findings suggest that digital strategy should be designed around structural priorities rather than around the isolated adoption of tools. The key issue is not whether to digitalize, but which functions should remain under direct control, which should be modularized, and which should be coordinated through external platforms. Organizers with strong content, brand, or audience resources may benefit from retaining control over high-value functions, while using external partnerships selectively to extend reach and commercialization.

For platforms, sponsors, and service providers, the results highlight the importance of governance quality in collaborative arrangements. As digital upgrading increases interdependence, value creation depends less on simple transactional exchange and more on stable interfaces, data-sharing rules, and aligned incentives. Platform firms need to move beyond traffic provision and support deeper integration with event operations. Sponsors and service providers need clearer mechanisms for performance evaluation, resource coordination, and revenue sharing if they are to benefit from the restructuring process. The study also has implications for integrated operation across ticketing, membership, content, and data-based commercialization. These functions are increasingly connected rather than separate. Events that treat ticketing as a user-entry system, content as a conversion asset, and data as a coordinating resource are more likely to build sustainable commercial loops. This implies that digital management should be organized around connected user journeys and interoperable service systems rather than around fragmented departmental functions.

This study has several limitations. The case-based design and the event sample provide useful variation, but the generalizability of the findings remains bounded by the selected contexts and the availability of observable digital-upgrading episodes. The sample captures meaningful differences across event types, yet it may not fully represent smaller, less commercialized, or institutionally distinct events. There is also room to improve variable measurement and causal identification. Some constructs, especially value chain restructuring and data monetization, are inherently complex and must be approximated through composite indicators. Although the event-study and DID designs improve inference, residual endogeneity may still exist if unobserved strategic shifts coincide with digital upgrading. Future research can refine measurement with more granular transactional, behavioral, and contractual data, and can strengthen identification by exploiting more exogenous shocks or staggered policy settings.

This study extends existing theories in three ways. First, it enriches organizational boundary theory by showing that digital upgrading in sport events produces dynamic boundary adjustment rather than a simple shift between internalization and outsourcing. Second, it advances platform ecosystem theory by demonstrating how platforms reshape not only market access and distribution but also the allocation of value-creating activities and control positions within the industry chain. Third, it integrates dynamic capabilities with value chain research by explaining why similar digital technologies lead to different restructuring outcomes depending on organizations' ability to acquire, integrate, and commercialize digital resources.

The findings further suggest that digital transformation in sport events should be understood as a governance and capability reconfiguration process. The observed differences among content-led, platform-driven, service-integration, and data-asset extension paths are not random variations, but reflect different combinations of technological capability, organizational boundary choices, and ecosystem conditions. This theoretical explanation moves beyond describing what changes occur and clarifies why and when digital upgrading generates distinct value chain restructuring outcomes. The empirical findings should not be interpreted as indicating that digital upgrading always produces positive restructuring outcomes. Although the study identifies dominant restructuring paths, some cases demonstrate delayed transformation, partial adoption, and hybrid configurations where digital initiatives improve operational efficiency without fundamentally changing value creation structures. Future research should further investigate why similar digital investments generate different outcomes across organizations by examining failure mechanisms, governance conflicts, and capability gaps.

## 5 Conclusion

Digital upgrading is reshaping the sport event industry by changing how value activities are organized, coordinated, and captured. Based on a sequential mixed-method analysis of 12 sport event cases and 28 interviews, this study identifies four restructuring paths, including content-led, platform-driven, service-integration, and data-asset extension pathways. The findings show that digital upgrading is associated with changes in organizational boundaries, network structures, and value distribution patterns, with stronger effects observed in events characterized by higher platform capability and richer data resources. This study shows that digital upgrading in the sport event industry is not merely a process of technological optimization, but a structural force that reshapes organizational boundaries, reallocates value activities, and reconfigures the industry value chain. The evidence indicates that digital technologies influence the sport event industry through changes in control, connection, and value capture, leading to differentiated restructuring paths across content production, platform distribution, service integration, and data-asset extension. The results further suggest that deeper restructuring is more likely to occur in events with stronger platform capability, richer data assets, and more mature commercialization scenarios. By linking digital technology to boundary adjustment, activity reconfiguration, network evolution, and performance change, the study provides a more integrated explanation of how digital transformation reorganizes value creation in the sport event industry and offers practical implications for strategic governance and coordinated commercial development. Digital upgrading represents a complex restructuring process involving both opportunities and tensions. While digital technologies can enhance coordination and create new value capture opportunities, their outcomes depend on organizational capabilities, ecosystem relationships, and governance arrangements. Recognizing these contingent effects provides a more balanced understanding of how sport event industries evolve under digital transformation.

## Data Availability

The original contributions presented in the study are included in the article/Supplementary Material, further inquiries can be directed to the corresponding authors.
