# Supplementary material for: Research on the impact of digital upgrade of sports event industry on value chain reconstruction
Source: Front Sports Act Living. 2026 Sep 25;8:1859039. doi: 10.3389/fspor.2026.1859039 (PMC13651549; doi:10.3389/fspor.2026.1859039)
Supplement: Supplementary file 1 [file Datasheet1.pdf]

# Supplementary information

## Research on the Impact of Digital Upgrade of Sports Event Industry on Value Chain Reconstruction

Lide Su[1], ZhiXiang Wen[1]*,Te Bu[1]*

[1]College of Physical Education, Hunan Normal University, Changsha, China

*Corresponding author: ZhiXiang Wen, College of Physical Education, Hunan Normal University, No. 437 Lushan South Road, Yuelu District, Changsha City, Hunan Province, 410081, E-mail: 457254754@qq.com.Te Bu, College of Physical Education, Hunan Normal University, No. 437 Lushan South Road, Yuelu District, Changsha City, Hunan Province, 410081, E-mail: 616387696@qq.com

## 1. Qualitative Methodology

### 1.1 Case Selection and Research Design

The qualitative phase adopted a multiple-case comparative design. Twelve sport events were selected using theoretical sampling criteria, including event type diversity, digital upgrading intensity, commercialization characteristics, and data availability. The sample included professional events, comprehensive sport events, and mass participation events to capture different restructuring trajectories.

### 1.2 Interview Protocol and Data Collection

Semi-structured interviews were conducted with 28 participants, including event organizers (10), digital platform managers (7), sponsors and commercial partners (6), and service providers (5). Each interview lasted 45-70 minutes. Questions focused on digital upgrading objectives, organizational boundary changes, value activity redistribution, ecosystem collaboration, and commercialization mechanisms.

### 1.3 Coding Procedure and Reliability Assessment

The coding process followed three stages. Open coding generated first-order concepts from interview transcripts, reports, announcements, and operational documents. Axial coding grouped related concepts into second-order themes through

constant comparison. Selective coding integrated themes into higher-order categories linked to digital upgrading, organizational boundaries, and value chain restructuring. Two researchers independently coded the materials after training, and disagreements were resolved through discussion. The intercoder agreement coefficient was 0.86.

### 1.4 Emergence of Four Restructuring Paths

The four restructuring paths were derived through cross-case comparison. Content-led restructuring reflects internal strengthening of digital content capabilities. Platform-driven restructuring emerges when platforms become central coordinators of traffic and transactions. Service-integration restructuring reflects digital integration of ticketing and user services. Data-asset extension restructuring occurs when data resources are converted into commercial value.

### 1.5 Methodological Transparency

The use of multiple data sources, independent coding, intercoder agreement assessment, and systematic category development improves the credibility and replicability of the qualitative findings.

**Table S1. Case selection and characteristics**

| Case type | Number | Digital focus | Purpose |
|---|---|---|---|
| Professional events | 5 | Platform operation, media distribution, data services | Platform-driven restructuring |
| Comprehensive events | 3 | Integrated services and stakeholder coordination | Service integration |
| Mass participation events | 4 | User engagement and digital services | Content and data restructuring |

**Table S2. Interview statistics**

| Group | Number | Contribution |
|---|---|---|
| Event organizers | 10 | Strategic decisions and boundary adjustment |
| Platform managers | 7 | Platform coordination mechanisms |
| Sponsors | 6 | Commercial conversion |
| Service providers | 5 | Digital implementation |

**Table S3. Coding progression**

| Evidence | First-order codes | Second-order themes | Higher-order categories |
|---|---|---|---|
| Internal digital content teams | Capability retention; content control | Internal capability consolidation | Boundary contraction |

| | | | |
|---|---|---|---|
| Platform cooperation | Traffic coordination; platform dependence | Ecosystem collaboration | Platform-driven restructuring |
| Integrated ticketing systems | Service integration; interface standardization | Digital service coordination | Service-integration restructuring |
| Data-based sponsorship | User profiling; data monetization | Data commercialization | Data-asset extension |

**Table S4. Representative quotations**

| Path | Illustrative quotation | Interpretation |
|---|---|---|
| Content-led | Digital tools improve multi-channel content production. | Internal content capability enhancement |
| Platform-driven | Platforms connect events with audiences and sponsors. | Platform coordination role |
| Service-integration | Ticketing and membership are integrated digitally. | Modular service operation |
| Data-asset extension | User data support sponsorship evaluation. | Data-based value creation |

## 2. Qualitative Validation Evidence

### 2.1 Evidence Traceability and Audit Trail

To enhance qualitative validation, this supplementary material provides an evidence traceability framework linking empirical materials, coding results, and theoretical interpretations. Each restructuring path was derived from multiple sources, including interviews, official documents, operational records, and industry reports, rather than isolated observations.

### 2.2 Path Derivation Logic

The four restructuring paths were derived through cross-case comparison of digital change locations, organizational responses, and value capture changes. Cases were examined according to whether digital upgrading primarily affected content production, platform coordination, service integration, or data commercialization. A restructuring path was identified only when similar patterns appeared across multiple cases.

### 2.3 Qualitative Validation Procedures

Source triangulation was conducted by comparing interview materials with documentary evidence. Negative cases were reviewed to examine situations where digital upgrading produced limited restructuring effects. Emerging interpretations were repeatedly checked against original materials to ensure that theoretical categories

remained grounded in empirical observations.

**Table S5. Evidence chain from empirical observations to restructuring paths**

| Empirical evidence | Observed change | Analytical interpretation | Restructuring path |
|---|---|---|---|
| Digital content teams and multi-channel production | Content control increased | Core capability retained within organizer boundary | Content-led restructuring |
| Platform cooperation for traffic and user access | Platform coordination expanded | External ecosystem collaboration strengthened | Platform-driven restructuring |
| Integrated ticketing and membership systems | Service processes connected | Activities became modular and interoperable | Service-integration restructuring |
| User analytics and sponsor data systems | Data became commercial resource | Value capture shifted toward data-enabled activities | Data-asset extension restructuring |

**Table S6. Representative quotations supporting path derivation**

| Category | Representative quotation | Interpretation |
|---|---|---|
| Boundary adjustment | Digital systems allow us to retain control of core content while connecting external partners. | Selective boundary adjustment |
| Platform coordination | Platforms provide audience access, data support, and commercial connections. | Platforms become ecosystem coordinators |
| Service integration | Ticketing, membership, and user services are managed through connected systems. | Digital interfaces integrate activities |
| Data commercialization | Data analysis supports sponsor evaluation and new service design. | Data becomes a value creation resource |

## 3. Network Construction and Measurement Procedures

### 3.1 Network construction framework

The network analysis was conducted to examine how digital upgrading changes relationships among actors in the sport event value chain. The network unit was defined as an organizational actor. Actors included event organizers, digital platforms, media organizations, ticketing providers, sponsors, technology service firms, and data service providers. Actor identification was based on official event documents, annual reports, partnership announcements, sponsorship disclosures, platform operation materials, and interview records. The final dataset included 72 actors across 12 sport event cases.

### 3.2 Tie identification and coding procedure

Network ties were identified through observable collaboration and transaction relationships. A tie was coded when two actors had documented interaction in content production, digital distribution, ticketing integration, sponsorship activation, data exchange, or platform cooperation. Two researchers independently coded actor relationships and verified ambiguous cases through additional evidence. The final intercoder agreement coefficient was 0.89.

### 3.3 Binary and weighted network construction

Both binary and weighted networks were constructed. In the binary network, $a_{ij} = 1$ indicates the existence of a relationship between actor i and actor j, while $a_{ij} = 0$ indicates no direct relationship. The weighted network captures relationship intensity through cooperation frequency, transaction scale, and collaboration diversity. A three-level weighting scheme was applied, where 1 represents occasional cooperation, 2 represents repeated cooperation, and 3 represents multi-dimensional strategic cooperation.

### 3.4 Data sources and reliability control

Network construction integrated multiple sources, including official announcements, annual reports, sponsorship materials, industry reports, platform cooperation records, and interview evidence. Documentary sources were used to identify visible relationships, while interviews were used to verify collaboration mechanisms and hidden linkages. Cross-source triangulation was conducted before generating the final adjacency matrices.

### 3.5 Network measurement indicators

Network density was used to evaluate overall connectivity. Degree centrality and betweenness centrality were used to identify influential actors and brokerage positions. Collaborative embeddedness was measured through weighted relationship intensity and repeated interactions among actors.

$$D = L / [N(N-1)]$$

where L represents the number of observed ties and N represents the number of actors.

$$C_D(i) = \Sigma_j a_{ij} / (N-1)$$

where $a_{ij}$ represents the relationship between actor i and actor j.

$$C_B(i) = \Sigma_{j<k} g_{jk}(i) / g_{jk}$$

where $g_{jk}$ is the number of shortest paths between actors j and k, and $g_{jk}(i)$ is the

number of paths passing through actor i.

### 3.6 Network construction workflow

The construction procedure consisted of five steps: actor identification, relationship extraction, tie coding, adjacency matrix generation, and network metric calculation. This procedure ensures that network indicators reflect observable changes in industrial relationships rather than subjective assessments.

**Table S7. Network actor categories and data sources**

| Actor category | Examples | Primary data sources | Network role |
|---|---|---|---|
| Event organizers | League operators and event committees | Reports and interviews | Core coordinator |
| Digital platforms | Media and service platforms | Partnership announcements | Traffic and connection hub |
| Ticketing providers | Ticketing and membership systems | Service documents | User access node |
| Sponsors | Commercial partners | Sponsorship materials | Commercial conversion node |
| Data service firms | Technology and analytics providers | Technical documents and interviews | Data capability provider |
